# Supplementary material for: The NELF pausing checkpoint mediates the functional divergence of Cdk9
Source: Nat Commun. 2023 May 13;14:2762. doi: 10.1038/s41467-023-38359-y (PMC10182999; doi:10.1038/s41467-023-38359-y)
Supplement: Supplementary file 1 — Supplementary Information [file 41467_2023_38359_MOESM1_ESM.pdf]

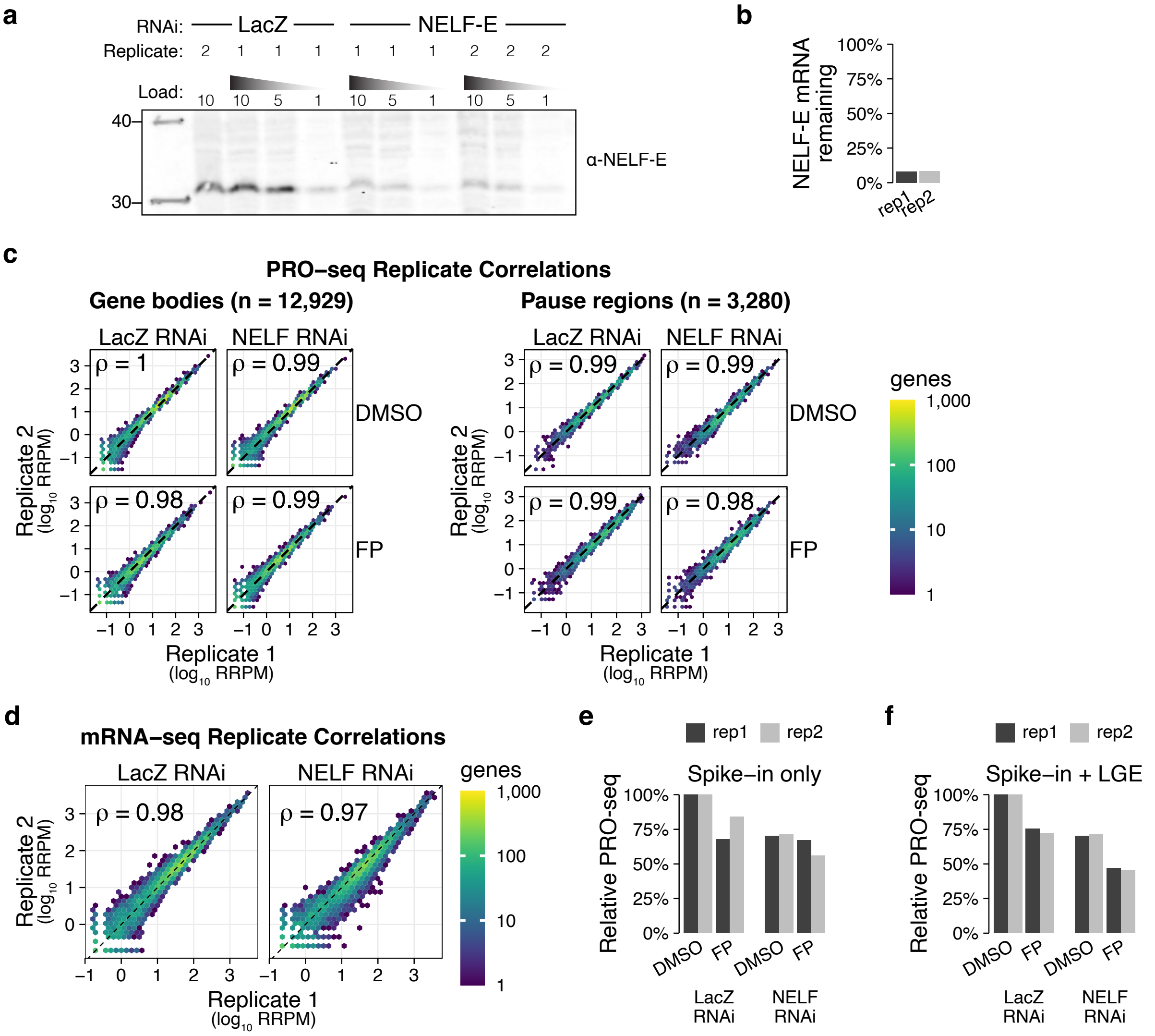

Supplementary Figure 1. Overview of PRO-seq and mRNA-seq in NELF-depleted S2 cells.

a) Western blot targeting NELF-E, shown for S2 cells treated with dsRNA targeting NELF-E or LacZ as a control. Samples from 2 replicates are shown, some with dilution series to provide estimates of quantitative changes. We estimate ~15-30% of NELF-E remains. See Supplementary Fig. 7 for uncropped image.

b) NELF-E mRNA knockdown efficiency measured using mRNA-seq. For each replicate, the ratio of spike-in normalized reads for NELF-E mRNA in NELF RNAi vs. LacZ RNAi is plotted.

c) PRO-seq read counts for each replicate are plotted against one another for each condition, and Spearman correlations are indicated. Gene bodies are the 12929 “consensus” regions (see Methods; Supplemental Materials and Methods) which begin 300 bp downstream of the most downstream annotated TSS and end 300 bp upstream of the most upstream annotated CPS, and all regions longer than 100 bp are kept. Pause regions are within 100 bp downstream of any PRO-cap-filtered TSS associated with an annotated promoter (see Methods; Supplementary Materials and Methods).

d) Replicate correlations of mRNA-seq data. For all mRNA-seq analyses, only reads mapping within 500 bp upstream of an annotated CPS are counted, and only genes analyzed for gene body PRO-seq are used (n=12876 genes with n=14836 CPS sites; see Methods; Supplementary Materials and Methods).

e) Within each replicate, the total number of PRO-seq reads mapping to the *Drosophila* genome is divided by the total number of reads mapping to the mouse spike-in. These ratios are then divided by that of the replicate-matched negative control (LacZ RNAi +DMSO) to show the relative quantity of Drosophila PRO-seq material obtained.

f) The DMSO conditions are the same as in panel c, but the FP conditions for each RNAi treatment are normalized to the replicate-matched DMSO condition using the reads mapping to the 3' ends of long genes (Long Gene End normalization, LGE). This additional normalization is used as it reduces replicate variance in the spike-in normalization for the FP conditions, likely resulting from a technical inaccuracy in cell counting or the addition of the spike-in. A later experiment validated this correction approach and our overall spike-in normalization strategy (Supplementary Fig. 5).

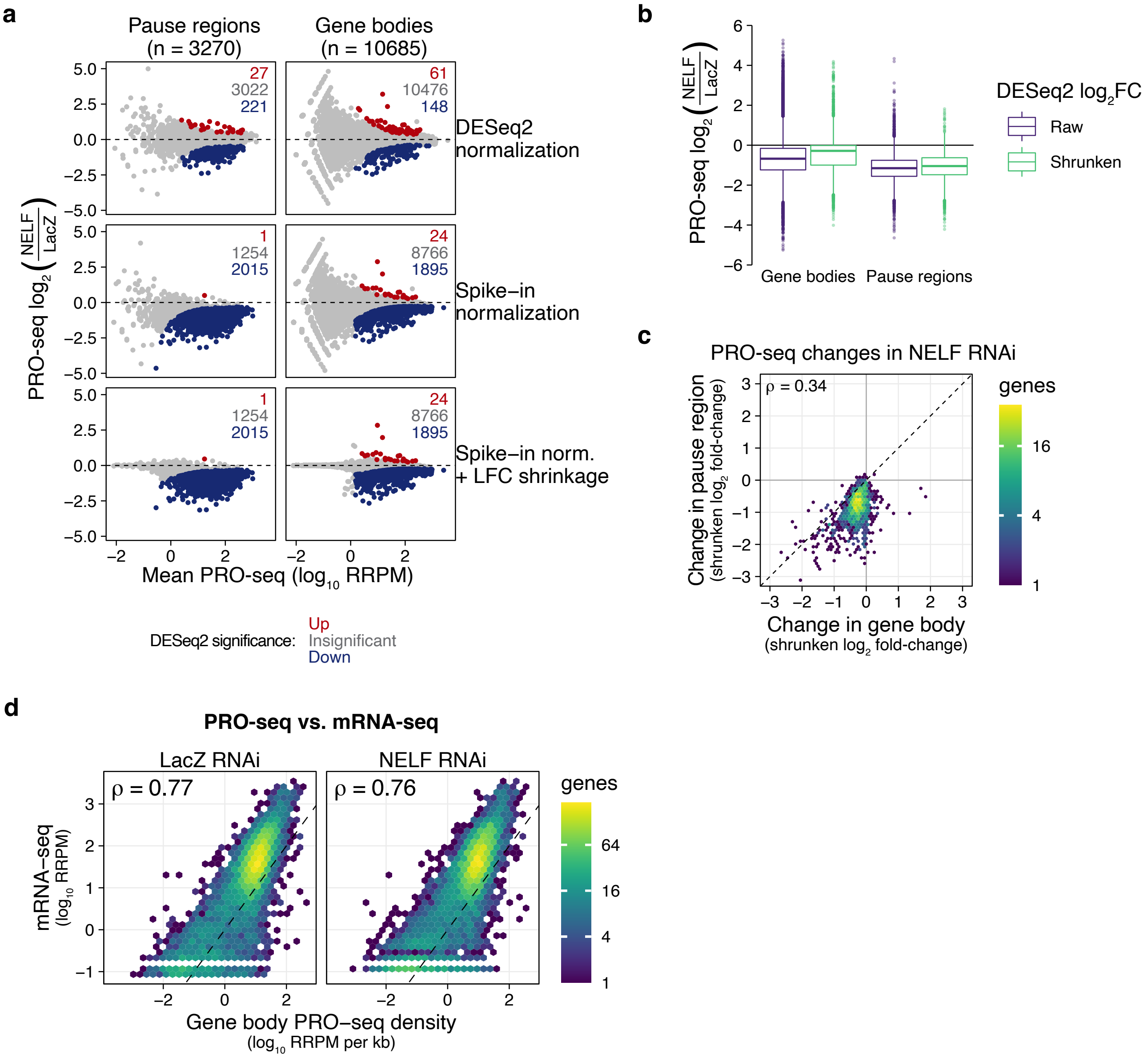

Supplementary Figure 2. Gene-specific changes in PRO-seq and mRNA-seq following NELF depletion.

a) MA plots associated with DESeq2 differential gene expression analysis comparing NELF RNAi to the LacZ RNAi control. The plots demonstrate the importance of spike-in normalization as compared to typical DESeq2 normalization, which assumes no global change in transcription. Log fold-change (LFC) shrinkage (from the R package *apeglm*) does not affect DESeq2 significance.

b) Distributions of LFCs with and without shrinkage for n=3280 pause regions and n=12929 “consensus” gene bodies (see Methods). “Raw” refers to non-shrunken LFCs. Boxes show median + interquartile range (IQR), whiskers extend to 1.5x IQR.

c) For a matched set of filtered pause regions and their corresponding gene bodies (n=1434), the shrunken LFC in NELF RNAi vs. the LacZ control are compared. A gene with no change in pausing index (the ratio of Pol II density in the pause region vs. the downstream gene body region) would be on the dashed x=y line; genes with decreased pausing indices are below the line.

d) Comparison of PRO-seq gene body density to mRNA-seq. PRO-seq gene body reads are normalized to the length of the analyzed region, while no length normalization is necessary for mRNA-seq given its 3' RNA-seq methodology (see Methods). Spearman correlations are shown. Even without correcting for gene length and the decreasing Pol II density throughout the first several kilobases of gene body (Fig. 2b), Pearson's R^2 indicates gene body PRO-seq density accounts for 67% and 65% of variance in mRNA-seq read counts in the respective conditions.

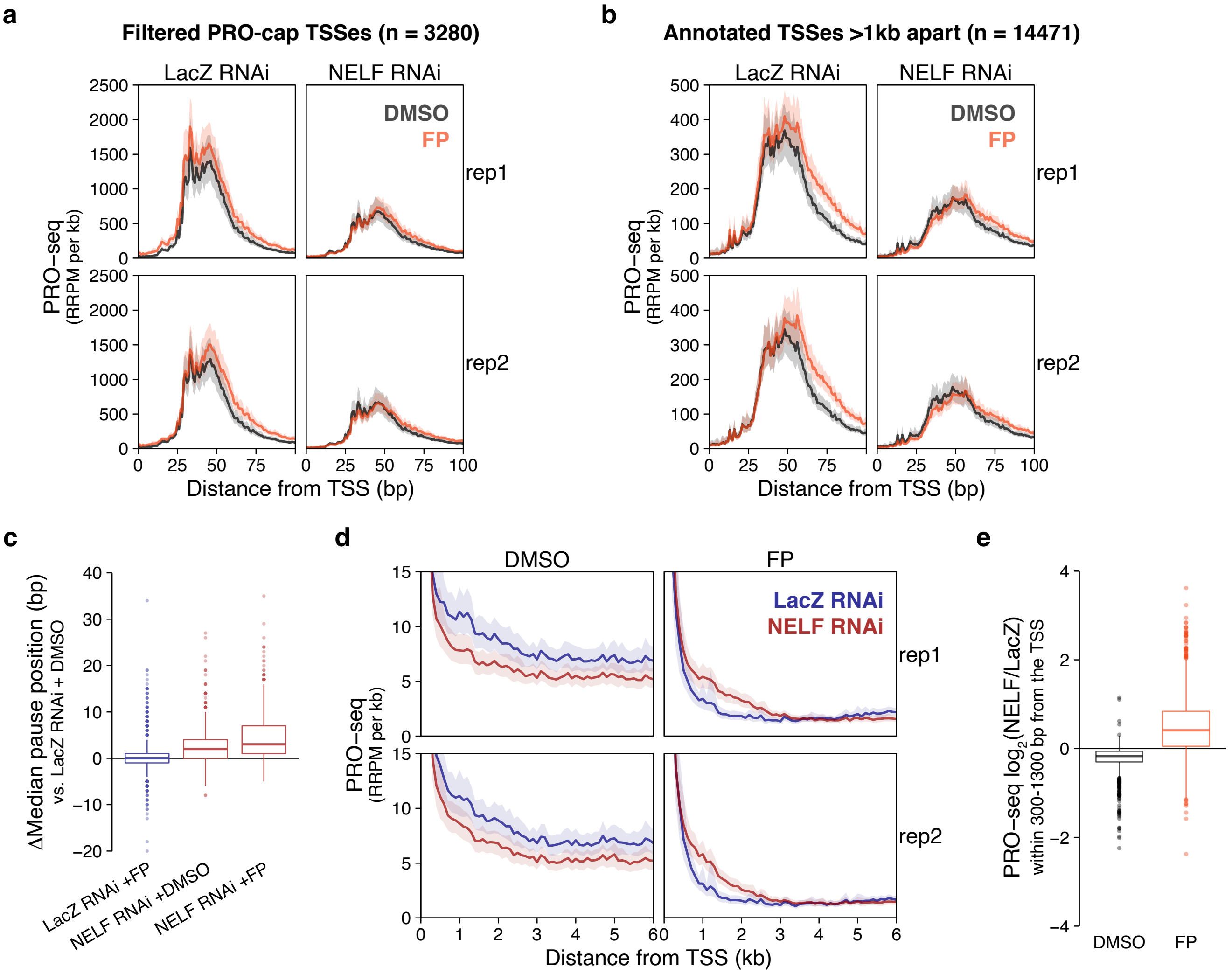

Supplementary Figure 3. PRO-seq profile changes in NELF RNAi and Flavopiridol-treated S2 cells.

a) Metagene profiles showing bootstrapped mean PRO-seq (+75% confidence intervals) for each condition and for each replicate separately. These plots are the full set of PRO-cap filtered pause regions (see Methods).

b) Metagene profile plots as in panel a, but over a largely unfiltered set of annotated TSSes, showing that the observed phenotypes are not unfairly enriched by our stringent TSS filtering.

c) For each condition, the median Pol II position within the pause region (TSS to TSS+100) is calculated by finding the position (distance from TSS) which evenly divides the PRO-seq signal within that region such that 50% of signal is upstream and 50% is downstream. For each gene, the median position in each plotted condition is subtracted from that found for the LacZ RNAi +DMSO control, and the boxplots show those distributions. From the n=1560 filtered pause regions that have matching filtered gene bodies, only pause regions with an average PRO-seq of >5 RRPM in LacZ RNAi +DMSO samples were used (n=1259). Boxes show median + IQR, and whiskers extend to 1.5x IQR or the most extreme value (whichever is closer to the median).

d) PRO-seq metagene profiles like those in Fig. 1c, but showing each replicate separately.

e) For each drug treatment condition, PRO-seq shrunken log2 fold-changes (LFCs) for NELF vs. LacZ RNAi are found within 300-1300 bp from the TSS for filtered genes >1.6kb in length (n=1243). Out of those genes, n=993 (80%) have shrunken LFCs >0 in the NELF RNAi +FP vs. LacZ RNAi +FP. Boxplotting statistics are defined the same as in panel c.

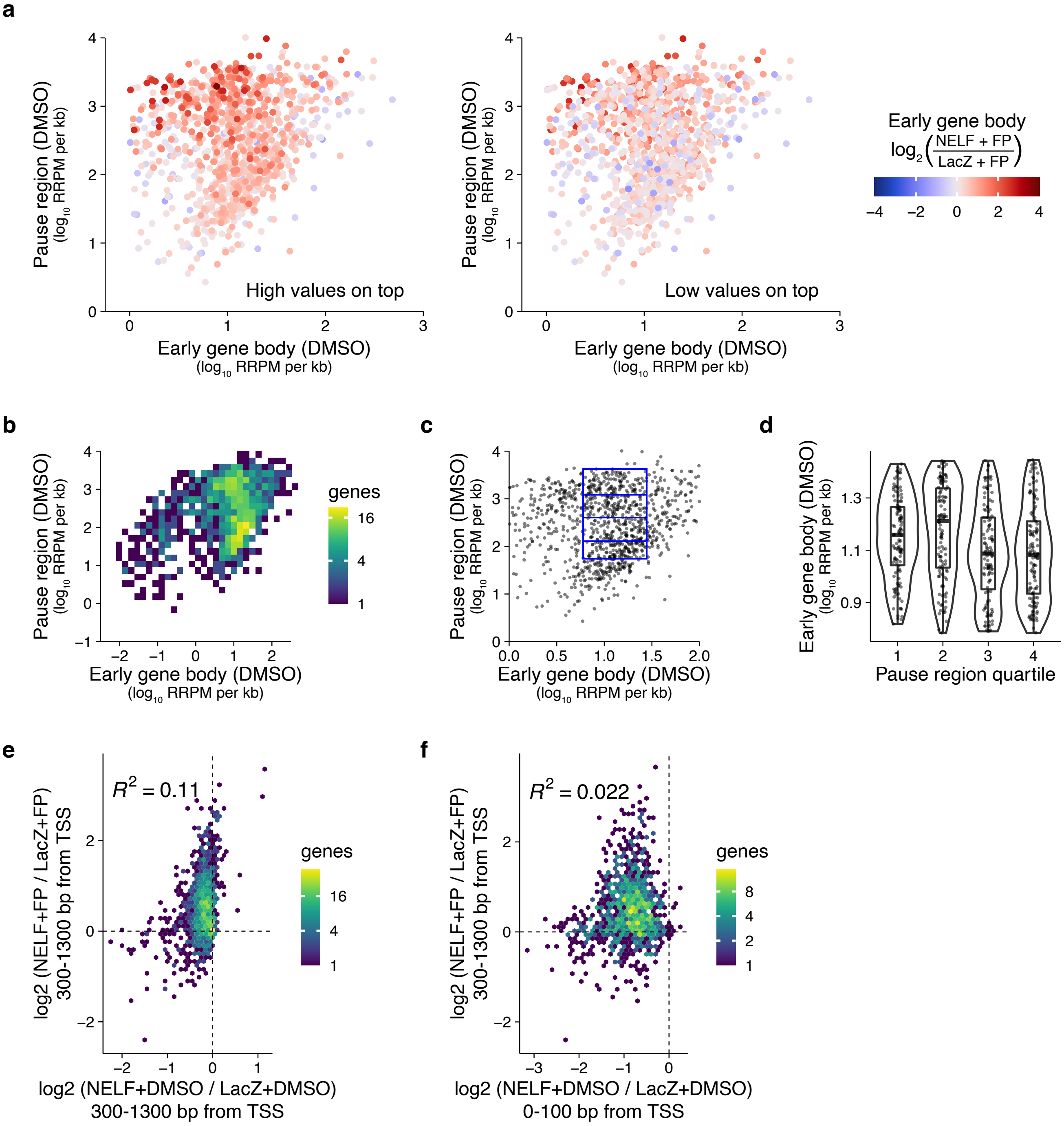

Supplementary Figure 4. Related to Figure 3.

a) Related to Fig. 3b, x and y axes show the relationship between PRO-seq signal (arithmetic mean across the LacZ and NELF RNAi DMSO conditions) in the early gene body (TSS+300 to TSS+1300 bp) and in the pause region (TSS to TSS+100) for filtered genes >1.6kb (n=1243) which have at least 1 RRPM/kb in both regions (n=1045). Colors (see legend) are based on shrunken log fold-changes (LFCs). To address overplotting, the same data is plotted twice: with highest LFCs on top (left) and with lowest LFCs on top (right).

b) Related to Fig. 3b, the number of genes within each 2D bin is shown.

c) The same scatterplot as in panel a, but with blue boxes indicating the subset of genes used for the metagene profiles in Fig. 3c. Genes with early gene body PRO-seq 6-28 RRPM/kb and pause region 54-5000 RRPM/kb (n=492) are divided into quartiles by pause signal.

d) For genes in panel c, the distributions of early gene body PRO-seq density are shown for each pausing quartile. Boxes show median + IQR, whiskers extend to most extreme value or to 1.5x IQR (whichever is closer to the median).

e) For filtered genes >1.6kb in length (n=1243), the shrunken LFC within 300-1300 bp from the TSS following NELF-depletion (x-axis) is plotted against the aberrant early gene body density, quantified as the shrunken LFC in NELF vs. LacZ RNAi FP conditions (y-axis). This shows that the gene body response to NELF depletion has a weak relationship to the quantity of abnormal Pol II density within the early gene body observed following FP treatment in NELF-depleted cells.

f) Similar to panel e, except the x-axis shows the shrunken LFC within pause regions following NELF-depletion. This shows that the pause region response to NELF depletion has no relationship to the quantity of abnormal Pol II observed in the early gene body following FP treatment in NELF-depleted cells.

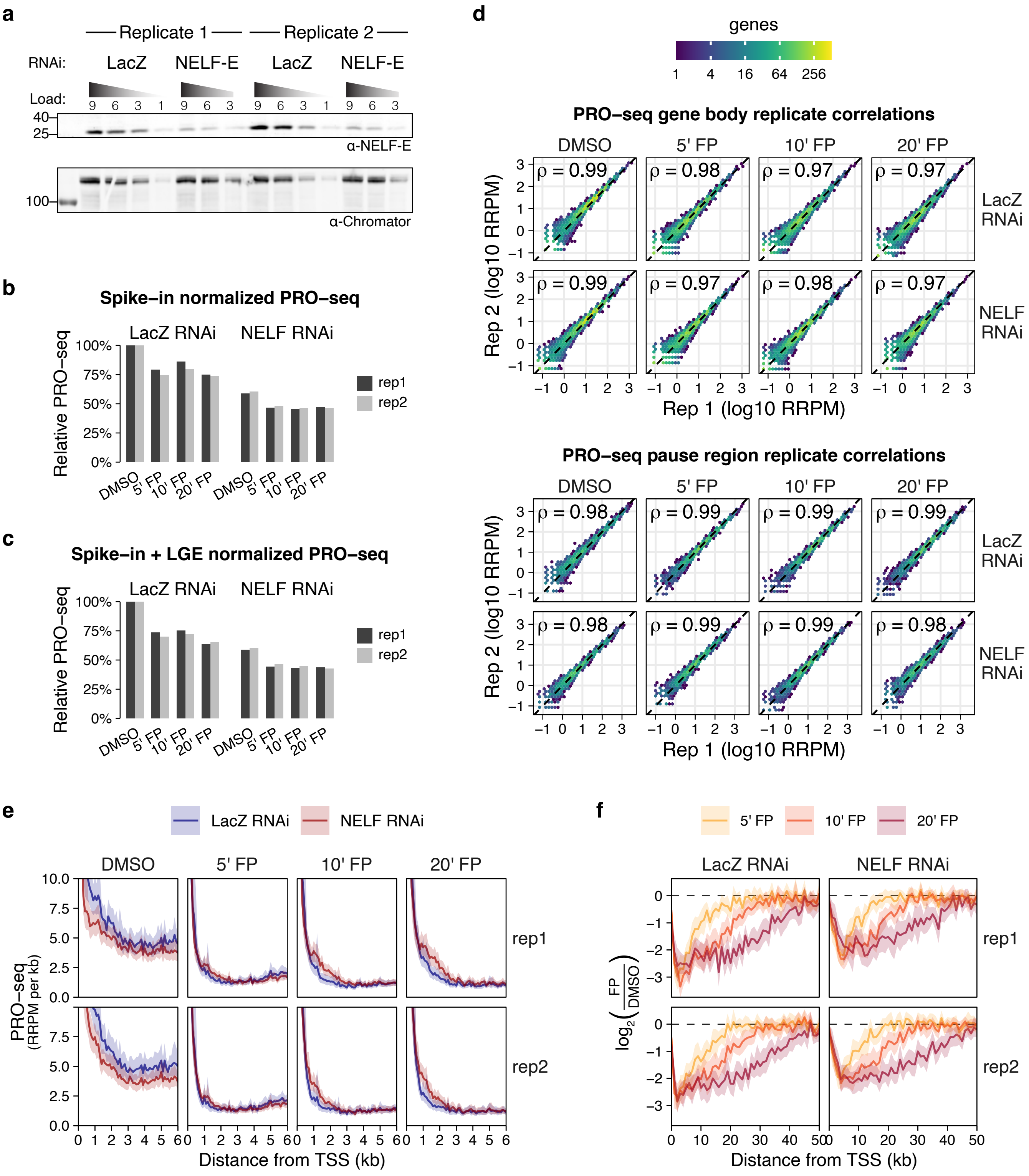

Supplementary Figure 5. Related to the Flavopiridol time-course PRO-seq in NELF vs. LacZ RNAi S2 cells.

a) Western blot showing NELF-E knockdown efficiency in both replicates of DMSO-treated NELF and LacZ RNAi S2 cells associated with the FP time-course PRO-seq experiments. Anti-Chromator staining provided for reference. We estimate roughly 15-30% of NELF-E remains. This experiment was performed twice. Size markers do not appear in the NELF-E channel; see Supplementary Fig. 7 for uncropped Westerns.

b) Same plot as was done for the first experimental batch (Supplementary Fig. 1c): within each replicate, the total number of PRO-seq reads mapping to the Drosophila genome is divided by the total number of reads mapping to the mouse spike-in. These ratios are then divided by that of the replicate-matched negative control (LacZ RNAi +DMSO) to show the relative quantity of PRO-seq material obtained in each condition.

c) As in Supplementary Fig. 1d, the DMSO conditions are the same as in panel b, but the FP conditions for each RNAi treatment are normalized to the replicate-matched DMSO condition using the reads mapping to the 3' ends of long genes (Long Gene End normalization, LGE). In this case, the long gene-end normalization factors are very similar to the spike-in normalization factors for each FP-treated condition.

d) As in Supplementary Fig. 1e, replicate correlations of PRO-seq read counts for each condition within both unique annotated gene bodies (n=12929) and PRO-cap-filtered pause regions (n=3280).

e) Same as Fig. 4g (bootstrapped mean + 50% CI within 100 bp bins), but PRO-seq metagene profiles are shown for each replicate.

f) Same as Fig. 4e-f (bootstrapped mean + 50% CI within 1kb bins), but metagene profiles for each replicate are shown (each showing the gene-wise log fold-changes in each FP condition vs. DMSO for that replicate).

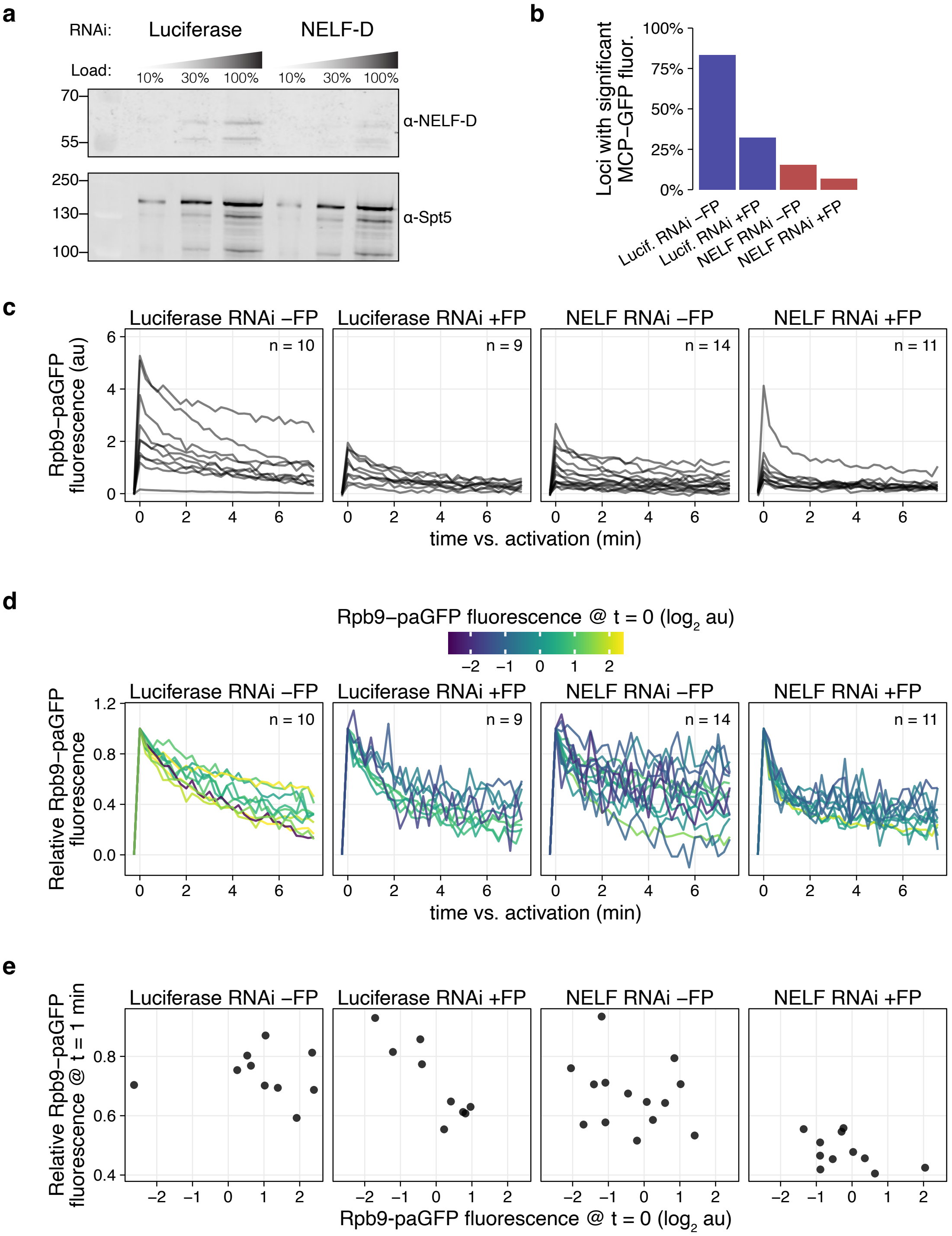

Supplementary Figure 6. Related to live-imaging of Pol II in NELF-depleted salivary glands.

a) Western blot validation of RNAi fly lines, showing NELF-D knockdown in dissected salivary glands. Serial dilutions for each are loaded (100% = 20 μg). Based on the serial dilutions, we estimate <30% of NELF-D remains. Anti-Spt5 staining is included for reference, but not relied on as a quantitative loading control given its role in transcription by Pol II. Both proteins appear as doublets. This experiment was performed twice.

b) For each RNAi and drug treatment condition (x-axis), the total fraction of mCherry-positive salivary glands with MCP-GFP signal present above background levels is shown. MCP-GFP binds to the MS2 stem-loops that form within the nascent RNA of the Hsp70 transgene (~1.3-2.5kb downstream of the TSS, see Fig. 6a).

c) Lineplots showing Rpb9-paGFP fluorescence intensity for individual (single locus) photoactivation time-series. Background-subtracted GFP fluorescence intensity is integrated within a 3D region slightly larger than the Hsp70 transgene locus (identified via LacI-mCherry).

d) For each time-series sample plotted in panel c, relative fluorescence intensity is plotted (normalized as 0 before photoactivation and 1 immediately after at t=0). For each individual time-series, line color indicates the background-subtracted fluorescence intensity (log2) immediately after photoactivation (t=0 in panel c).

e) Relationship between photoactivation strength (x-axis; background subtracted fluorescence signal as in panel c) and the relative quantity decayed in 1 minute (y-axis; as in panel d).

a

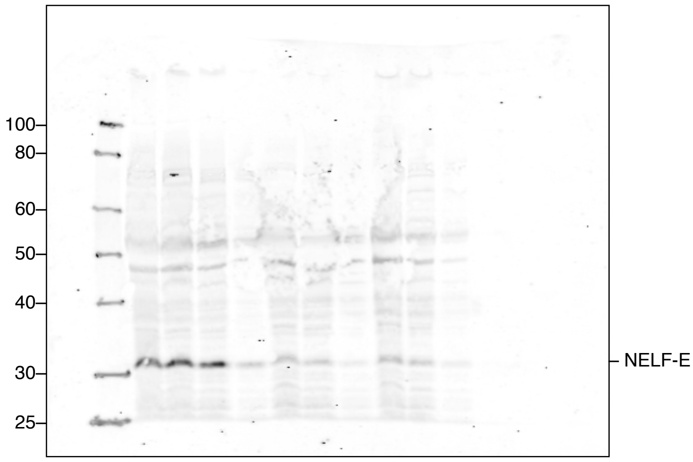

b

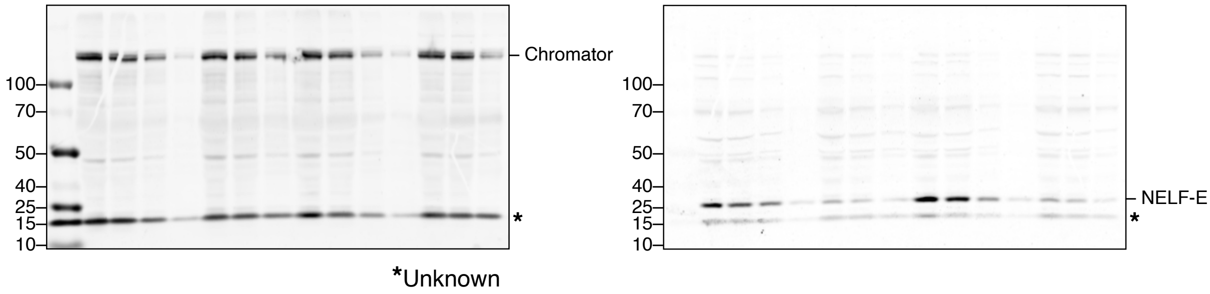

c

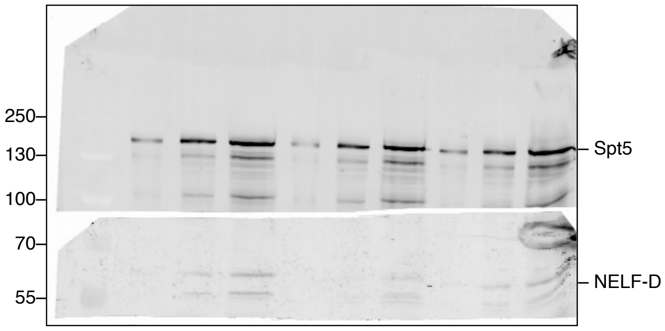

Supplementary Figure 7. Uncropped Western blots.

a) Uncropped Western blot from Supplementary Fig. 1a.

b) Uncropped Western blot from Supplementary Fig. 5a.

c) Uncropped Western blot from Supplementary Fig. 6a. Right 3 lanes show a NELF-E RNAi line, which was not used in any other experiments this study.

Supplementary Materials and Methods

PRO-seq statistics:

exp	sample	Total reads	Pass filter	Non rRNA	Unique align	Exp reads	Spike reads	NF spike-only	NF adjust
1	LacZKD_DMSO_rep1	28621545	24486014	21305287	15448661	15431880	16781	0.0648	0.0648
1	LacZKD_DMSO_rep2	57313389	48435833	42673981	30904394	30868025	36369	0.0324	0.0324
1	LacZKD_FP_rep1	37024799	28440702	23463837	16627236	16600591	26645	0.0408	0.0505
1	LacZKD_FP_rep2	29120528	24376588	20073791	14357523	14337436	20087	0.0587	0.0557
1	NELFeKD_DMSO_rep1	34883383	30002593	25639867	17887325	17859670	27655	0.0393	0.0393
1	NELFeKD_DMSO_rep2	29016470	24892869	21579810	14941194	14916553	24641	0.0478	0.0478
1	NELFeKD_FP_rep1	25214785	20057641	16015734	11015741	10997898	17843	0.0609	0.0463
1	NELFeKD_FP_rep2	38057658	31718644	25887789	17447182	17410615	36567	0.0322	0.0283
2	LacZKD_DMSO_rep1	11434214	9130155	8433209	6031108	6025855	5253	0.166	0.166
2	LacZKD_DMSO_rep2	12158806	10919851	9733842	6812291	6809788	2503	0.1468	0.1468
2	LacZKD_FP_05min_rep1	13769839	12113605	11024897	7289838	7283118	6720	0.1297	0.1088
2	LacZKD_FP_05min_rep2	14435845	13134848	11356439	7593499	7589628	3871	0.095	0.0983
2	LacZKD_FP_10min_rep1	21819969	18579894	16658312	11256006	11247123	8883	0.0981	0.0764
2	LacZKD_FP_10min_rep2	11992205	10760556	9049527	6133610	6131219	2391	0.1537	0.1301
2	LacZKD_FP_20min_rep1	18517152	15815060	12941804	8372596	8363618	8978	0.0971	0.0894
2	LacZKD_FP_20min_rep2	17206323	15359749	11391786	7425064	7421684	3380	0.1087	0.0996
2	NELFeKD_DMSO_rep1	17194043	14520878	12844662	8590739	8578013	12726	0.0685	0.0685
2	NELFeKD_DMSO_rep2	13982089	12453918	10570663	7195173	7190785	4388	0.0838	0.0838
2	NELFeKD_FP_05min_rep1	12312517	8370178	7238227	4430681	4422333	8348	0.1044	0.1056
2	NELFeKD_FP_05min_rep2	8939194	6625916	5493489	3503597	3500915	2682	0.137	0.1363
2	NELFeKD_FP_10min_rep1	14996753	12551462	10515900	6645826	6635603	10223	0.0853	0.0689
2	NELFeKD_FP_10min_rep2	13826267	12295271	9698741	6314310	6310459	3851	0.0954	0.0732
2	NELFeKD_FP_20min_rep1	12269562	5293483	4123252	2502093	2497218	4875	0.1788	0.1878
2	NELFeKD_FP_20min_rep2	18503535	11133933	8201023	5164988	5161456	3532	0.1041	0.0897

[*] "Pass filter" is mostly filtering by insert length. But for experiment 1, "pass filter" also includes UMI-based deduplication (see Methods).

PRO-cap statistics

Sample	Total reads	Pass filter	Non rRNA	Unique align	Exp reads	Spike reads
LacZKD	27276810	26140336	25095693	13755367	13731462	23905
LacZKD_noTAP	42886855	39706955	34727710	22774243	22738831	35412

RNA-seq statistics

Sample	Total reads	Pass filter	Unique align	Non-duplicate	Exp reads	Spike reads	NF
LacZKD_rep1	14153386	13832942	10816605	5214216	5156155	58061	0.1939
LacZKD_rep2	14148847	13802217	10425394	4877994	4817990	60004	0.2076
NELFeKD_rep1	11911524	11587101	8528265	4360074	4312775	47299	0.2381
NELFeKD_rep2	12264885	11907046	8713538	4066973	4015509	51464	0.242

[*] For RNA-seq, UMI-based deduplication occurs after alignment (see Methods).

Primers for generating DNA templates for IVT of dsRNA

LacZ (F)	GAATTAATACGACTCACTATAGGGAGAGATATCCTGCTGATGAAGC
LacZ (R)	GAATTAATACGACTCACTATAGGGAGAGCAGGAGCTCGTTATCGC
NELF-E (F)	GAATTAATACGACTCACTATAGGGAAGGCACTGCAAGCGCACAAGGCGCCC
NELF-E (R)	GAATTAATACGACTCACTATAGGGACTTCATCGTATTGAACCATCTCGCGG

S2 cell counts following RNAi

Experiment*	Replicate	dsRNA	Cells x 10^6 / mL
1	1	LacZ	4.95
1	1	NELF-E	4.78
1	2	LacZ	4.94
1	2	NELF-E	4.76
2	1	LacZ	5.15
2	1	NELF-E	5.44
2	2	LacZ	5.14
2	2	NELF-E	5.09

[*] Experiment 2 is the Flavopiridol time-course experiment

Average cell count ratio of NELF / LacZ RNAi (mean ± se): 0.994 ± 0.0217

TSS refinement and stringent gene list filtering for basepair-resolution analysis

For most analyses in this paper, we use a gene list in which we selected or adjusted the annotated TSS of each annotated gene using our PRO-cap data. For this highly filtered gene list, we prioritized having a single high-confidence TSS for a given transcription unit (particularly for the pause and early gene body), such that we could treat the PRO-seq signal distribution as “distance transcribed” with little interference from alternative TSSes in the region or from readthrough transcription originating from sites further upstream.

To make this stringent gene list, we divided 34689 annotated transcripts according to their 17660 associated gene annotations, and for each gene we chose the most upstream CPS (cleavage and polyadenylation signal) as our annotated 3’ end, and we eliminated any gene for which an annotated CPS exists upstream of any annotated TSS. To find PRO-cap TSSes that appear consistent with that annotation (as opposed to something like an intragenic enhancer, for example), we considered PRO-

cap sites with at least 3 adjusted reads within [TSS-50, TSS+100] of any of that gene's annotated TSSes, and we selected a single annotated isoform as the annotation if its max PRO-cap site has more than 5x the signal of any other isoform, and we eliminated genes for which this was not the case. Using the max PRO-cap TSS of those selected isoforms, we selected a total of 7328 unique TSSes (at this stage filtering only for annotations at least 100 bp in size). As TSS usage within a given promoter region can vary – with some being focused at a single site while others are more diffuse across several – we filtered out PRO-cap TSSes for which a higher signal site is found within 100 bp (leaving n = 6957 TSSes), and took only those containing >50% of all PRO-cap signal within [TSS-50, TSS+100] (n = 4939) and where no other PRO-cap site within that region is within 2-fold of the max site (leaving n = 4347). Then, taking only annotations at least 800 bp in length (n = 3881), we filtered genes for readthrough transcription, accepting only those for which the PRO-seq signal 300-500 bp downstream of the TSS is more than 4x that within 300-100 bp upstream (n = 2272). For PRO-seq signal, we used the first (non-time-course) experiment's LacZ RNAi +DMSO controls. Finally, because PRO-seq reads originating from the TSS are never mappable before TSS+15, and because batch differences could exist between the PRO-cap and PRO-seq data (which are not matched), we selected only TSSes for which the PRO-seq signal in [TSS+15, TSS+30] is >4x that within [TSS, TSS+15] for a final of 1560 PRO-cap TSSes (which are associated with annotated gene TSSes and show a focused/unimodal pattern of initiation).

Throughout our analyses, we also verified that our gene list with stringent filtering for focused sites of initiation was not a significant determinant of our claimed phenotypes. We included a brief demonstration of this (**Supplemental Fig. 3a-b**).

<u>Less stringent filtering for separate analysis of promoter-proximal or gene body regions</u>

In some analyses, we did not require a usable gene body region to be matched to a promoter-proximal region, and we used only filtering by PRO-cap signal/concentration and readthrough transcription to identify promoter-proximal regions. Using the most upstream annotated TSS of the 17028 annotated genes longer than 100 bp (when the most downstream CPS is used), 7138 contain PRO-cap signal within [TSS-50, TSS+100]. Of those, we took those for which a single PRO-cap site within that region contained at least 40% of all local PRO-cap signal and we used that site as the TSS (n = 5599). We further filtered for upstream readthrough transcription by taking only genes for which PRO-seq within [TSS+300, TSS+500] was more than 4x that within [TSS-300, TSS-100] for a final set of 3280 PRO-cap TSSes.

For differential expression analyses, we also use a larger set of gene body regions without regard to TSS usage. For each annotated gene (n = 17660), we chose the "consensus" annotated gene body region as beginning TSS+300 bp from the most downstream annotated TSS and extending to CPS-300 bp of the most upstream annotated CPS. (We call this a "consensus" because the final region chosen is present in all annotated transcript isoforms). After taking only those with at least 100 bp of gene body remaining and eliminating any with overlaps, this left us with a final set of 12929 "consensus" gene body regions.

<u>Gene list filtering and read counting for RNA-seq data</u>

We used separate annotations for our 3' mRNA-seq, given that all reads of interest should be produced from priming off a polyA tail. We selected regions within 500 bp upstream of any

annotated CPS regions, and for *each individual annotated gene*, we produced a single comprehensive region or set of regions that maintain all of those regions but don't have any redundant sections (which would result in double-counting reads). However, any sections that overlap *multiple gene annotations* are considered ambiguous and those overlapping sections are removed from analysis. Of the 17194 annotated genes with at least 1 CPS-proximal region remaining, only those genes whose identifiers are also found in the 12929 consensus gene body regions above were used, for a final of n = 12876 genes (constituting a total of 14836 non-contiguous CPS-proximal segments).

Structure of Hsp70 transgene

The *Hsp70* transgene construct is composed of: (1) the 5'SCS *Hsp70* insulator element; (2) the 5' *Hsp70* sequence (from -252 to +765); (3) 24 MS2 loops; (4) the rest of the *Hsp70* gene; and (5) the 3' SCS *Hsp70* insulator sequence. The full sequence is included in a supplemental FASTA file. Approximately 1200bp upstream of the 5' end of the *Hsp70* transgene, 256 LacO binding sites are present to facilitate unambiguous targeting of the transgene by binding of transgenic mCherry-LacI. The entire construct (256xLacI-Hsp70MS2) was inserted by PhiC31 integration at attp16 (2R 53C4).
