# Supplementary material for: The NELF pausing checkpoint mediates the functional divergence of Cdk9
Source: Nat Commun. 2023 May 13;14:2762. doi: 10.1038/s41467-023-38359-y (PMC10182999; doi:10.1038/s41467-023-38359-y)
Supplement: Supplementary file 3 — Description of Additional Supplementary Files [file 41467_2023_38359_MOESM3_ESM.docx]

**Description of Additional Supplementary Files**

File name: Supplementary Data 1

Description: Hsp70 transgene sequence

File name: Supplementary Movie 1

Description: Luciferase RNAi

File name: Supplementary Movie 2

Description: Luciferase RNAi + 500 nM Flavopiridol

File name: Supplementary Movie 3

Description: NELF-D RNAi

File name: Supplementary Movie 4

Description: NELF-D RNAi + 500 nM Flavopiridol
