# Supplementary material for: The NELF pausing checkpoint mediates the functional divergence of Cdk9
Source: Nat Commun. 2023 May 13;14:2762. doi: 10.1038/s41467-023-38359-y (PMC10182999; doi:10.1038/s41467-023-38359-y)
Supplement: Supplementary file 9 — Reporting Summary [file 41467_2023_38359_MOESM9_ESM.pdf]

nature portfolio

Corresponding author(s): John T. Lis

Last updated by author(s): Mar 23, 2023

Reporting Summary

Nature Portfolio wishes to improve the reproducibility of the work that we publish. This form provides structure for consistency and transparency in reporting. For further information on Nature Portfolio policies, see our Editorial Policies and the Editorial Policy Checklist.

Statistics

For all statistical analyses, confirm that the following items are present in the figure legend, table legend, main text, or Methods section.

n/a	Confirmed	
☐	☒	The exact sample size (*n*) for each experimental group/condition, given as a discrete number and unit of measurement
☐	☒	A statement on whether measurements were taken from distinct samples or whether the same sample was measured repeatedly
☒	☐	The statistical test(s) used AND whether they are one- or two-sided<br>*Only common tests should be described solely by name; describe more complex techniques in the Methods section.*
☐	☒	A description of all covariates tested
☐	☒	A description of any assumptions or corrections, such as tests of normality and adjustment for multiple comparisons
☐	☒	A full description of the statistical parameters including central tendency (e.g. means) or other basic estimates (e.g. regression coefficient) AND variation (e.g. standard deviation) or associated estimates of uncertainty (e.g. confidence intervals)
☒	☐	For null hypothesis testing, the test statistic (e.g. *F*, *t*, *r*) with confidence intervals, effect sizes, degrees of freedom and *P* value noted<br>*Give P values as exact values whenever suitable.*
☒	☐	For Bayesian analysis, information on the choice of priors and Markov chain Monte Carlo settings
☒	☐	For hierarchical and complex designs, identification of the appropriate level for tests and full reporting of outcomes
☐	☒	Estimates of effect sizes (e.g. Cohen's *d*, Pearson's *r*), indicating how they were calculated

Our web collection on statistics for biologists contains articles on many of the points above.

Software and code

Policy information about availability of computer code

Data collection	Zeiss Zen Black was used for microscopic image acquisition.
Data analysis	Software utilized for data analysis, including version numbers and specific parameters for critical processing steps, are explicitly described in the Methods section and partially reproduced here: cutadapt v1.18, fastp v0.20.0, UMI-tools v1.0.0, prinseq_lite v0.20.4, bowtie2 v2.3.5, STAR v2.7.3a, R package BRGenomics v1.4.0 with DESeq2 v1.32.0 and apeglm v1.14.0

For manuscripts utilizing custom algorithms or software that are central to the research but not yet described in published literature, software must be made available to editors and reviewers. We strongly encourage code deposition in a community repository (e.g. GitHub). See the Nature Portfolio guidelines for submitting code & software for further information.

Data

Policy information about availability of data

All manuscripts must include a data availability statement. This statement should provide the following information, where applicable:

- Accession codes, unique identifiers, or web links for publicly available datasets
- A description of any restrictions on data availability
- For clinical datasets or third party data, please ensure that the statement adheres to our policy

All sequencing data for this work, including processed and normalized files, are deposited in GEO under accession number GSE211397. Microscopy data can be made available upon request.

Human research participants

Policy information about studies involving human research participants and Sex and Gender in Research.

Reporting on sex and gender	N/A. No human research participants.
Population characteristics	N/A. No human research participants.
Recruitment	N/A. No human research participants.
Ethics oversight	N/A. No human research participants.

Note that full information on the approval of the study protocol must also be provided in the manuscript.

Field-specific reporting

Please select the one below that is the best fit for your research. If you are not sure, read the appropriate sections before making your selection.

- [x] Life sciences
- [ ] Behavioural & social sciences
- [ ] Ecological, evolutionary & environmental sciences

For a reference copy of the document with all sections, see nature.com/documents/nr-reporting-summary-flat.pdf

Life sciences study design

All studies must disclose on these points even when the disclosure is negative.

Sample size	For imaging studies, a single sample is an imaging series on one dissected gland (each imaged only once). Sample size was determined according to the maximum number that could be attained within 3-4 hours of imaging, and performed on 3 separate days of imaging. For sequencing experiments, we targeted ~10 million S2 cells for PRO-seq as this was recommended in (doi) 10.1038/nprot.2016.086 and for 3' mRNA-seq we used ~2 million S2 cells as this was in the range recommended by the kit manufacturer Lexogen.
Data exclusions	No data was excluded, however glands were excluded from imaging studies if they drifted during imaging.
Replication	Sequencing experiments (PRO-seq, RNA-seq) are each done in 2 biological replicates (cell cultures separated by multiple passages), and treated as replicates in analysis. Additionally, a second distinct PRO-seq experiment (with its own replicates) was performed and independently analyzed to confirm major findings of the first experiment. All replicates of the reported experiments were successful and are presented in the manuscript. For imaging studies, all conditions were reproduced and imaged across 3 separate days, each of which is biologically independent (with separate parental crosses).
Randomization	For imaging, dissected glands were put into either DMSO or FP within alternating blocks of 3-5 glands. In each imaging session, samples from as many conditions as possible were imaged to obtain approximately the same number of samples for each condition in order to control for any variance associated with the timing or date of the experiment. No randomization was used in the sequencing experiments; all samples were handled in parallel (at nearly the same time) in a consistent manner by the same researcher.
Blinding	For MCP-GFP imaging experiments, the researcher was blinded to both RNAi and drug condition during imaging acquisition. For photoactivation imaging experiments, blinding is not relevant because the imaged molecules are not visible prior to image acquisition. For sequencing experiments, no blinding was used as there are no handling steps that are realistically subject to researcher bias.

Reporting for specific materials, systems and methods

We require information from authors about some types of materials, experimental systems and methods used in many studies. Here, indicate whether each material, system or method listed is relevant to your study. If you are not sure if a list item applies to your research, read the appropriate section before selecting a response.

Materials & experimental systems

n/a	Involved in the study	
[ ]	[x]	Antibodies
[ ]	[x]	Eukaryotic cell lines
[x]	[ ]	Palaeontology and archaeology
[ ]	[x]	Animals and other organisms
[x]	[ ]	Clinical data
[x]	[ ]	Dual use research of concern

Methods

n/a	Involved in the study	
[x]	[ ]	ChIP-seq
[x]	[ ]	Flow cytometry
[x]	[ ]	MRI-based neuroimaging

Antibodies

Antibodies used	Polyclonal antibodies derived from rabbit targeting Drosophila NELF-E and NELF-D (previously described in doi: 10.1101/gad.1091403 ; 10.1073/pnas.1000681107 ; 10.1128/MCB.05930-11) were gifts from David Gilmour (Pennsylvania State University). Guinea pig anti-dSpt5C was raised against residues 732-1054 for previous lab member Erik Andrulis by Pocono Farms (doi: 10.1101/GAD.844200) and is available upon request. Guinea pig anti-chromator was also made in house (#2654, bleed 2) by Pocono Farms and has been used previously (10.1101/gad.341768.120). LI-COR IRDye secondary antibodies used include donkey anti-rabbit 800CW (LI-COR 926-32213), donkey anti-guinea pig 800CW (LI-COR 926-32411), and donkey anti-guinea pig 680LT (LI-COR 926-68030).
Validation	Antibodies against NELF-E and NELF-D were previously validated using IP of FLAG-tagged transgenes in Drosophila and by RNAi knockdowns in Drosophila (10.1101/gad.1091403 ; 10.1073/pnas.1000681107 ; 10.1128/MCB.05930-11) and RNAi validation is again showed here in this manuscript. Anti-Spt5C was previously cross-validated using antibodies from 3 different species derived against 3 distinct segments of Drosophila Spt5, all of which identify the same bands on Western blots and colocalize with each other on chromatin as assayed with microscopy (10.1101/GAD.844200). As stated by the manufacturer, LI-COR antibodies are validated by immunoelectrophoresis and/or ELISA showing specific reactivity with target species IgG and not other serum components, non-target species serum, and the antibodies are targeted by ELISA and/or solid-phase adsorbed to show no cross reactivity against proteins from a number of other species.

Eukaryotic cell lines

Policy information about cell lines and Sex and Gender in Research

Cell line source(s)	No data from vertebrate cell lines are reported here, although mouse-derived embryonic fibroblast cells from ATCC (SCRC-1008, MEF (C57BL/6) [MEF-BL/6-1]) were used as exogenous spike-in. Drosophila melanogaster Schneider 2 (S2) cells were obtained from ATCC ("Shneider's Drosophila Line 2 [D. Mel. (2), SL2]", CRL-1963).
Authentication	Cell lines were not specifically authenticated in-house, but S2 cells match known growth and appearance characteristics, and sequencing data match with previous studies. Spike-in alignment also matches with the known species of origin.
Mycoplasma contamination	S2 cells stocks have tested negative for mycoplasma, but not the same stocks as those used in experiments. The mouse embryonic fibroblasts used for spike-ins were certified mycoplasma-free by the supplier and were not re-tested in our lab.
Commonly misidentified lines (See ICLAC register)	No commonly misidentified lines are used in this study.

Animals and other research organisms

Policy information about studies involving animals; ARRIVE guidelines recommended for reporting animal research, and Sex and Gender in Research

Laboratory animals	All flies strains used have their sources reported explicitly in the Methods section. These include lines publicly available from Bloomington DGRC: lines #6983, #7279, #31603. The UAS-RNAi[NELF-D] line is NELF-Di[8-2] from (doi) 10.1101/gad.1643208 and 10.1128/MCB.05930-11. UAS-Rpb9::paGFP, Sgs3-mCherry::LacI, and 256xLacO-Hsp70MS2 were generated and described in (doi) 10.1101/gad.231886.113. Drosophila melanogaster salivary glands were dissected from wandering third instar larvae.
Wild animals	No wild animals were used in this study.
Reporting on sex	This is not applicable to this study.
Field-collected samples	No field-collected samples were used in this study.
Ethics oversight	No ethical oversight or guidance was required as this study uses Drosophila melanogaster (insects) as a model system, and the only mammalian component is cultured mouse cells (used only as a spike-in control).

Note that full information on the approval of the study protocol must also be provided in the manuscript.
